# Overexpression of fatty acid synthase attenuates bleomycin induced lung fibrosis by restoring mitochondrial dysfunction in mice

**DOI:** 10.1038/s41598-023-36009-3

**Published:** 2023-06-03

**Authors:** Hyesun Shin, Shinhee Park, Jisu Hong, Ae-Rin Baek, Junehyuk Lee, Do-Jin Kim, An-Soo Jang, Su Sie Chin, Sung Hwan Jeong, Sung-Woo Park

**Affiliations:** 1 Division of Allergy and Respiratory Medicine, Department of Internal Medicine, Soonchunhyang University Bucheon Hospital, 170 Jomaru-ro, Wonmi-gu, Bucheon, 14584 Korea; 2 Department of Pathology, Soonchunhyang University Bucheon Hospital, Bucheon, 14584 Gyeonggi-do South Korea; 3 Department of Internal Medicine, Gachon University of Medicine and Science, Gil Medical Center, Incheon, Korea

**Keywords:** Mechanisms of disease, Animal biotechnology, Biomarkers

## Abstract

Proper lipid metabolism is crucial to maintain alveolar epithelial cell (AEC) function, and excessive AEC death plays a role in the pathogenesis of idiopathic pulmonary fibrosis (IPF). The mRNA expression of fatty acid synthase (FASN), a key enzyme in the production of palmitate and other fatty acids, is downregulated in the lungs of IPF patients. However, the precise role of FASN in IPF and its mechanism of action remain unclear. In this study, we showed that FASN expression is significantly reduced in the lungs of IPF patients and bleomycin (BLM)-treated mice. Overexpression of FASN significantly inhibited BLM-induced AEC death, which was significantly potentiated by FASN knockdown. Moreover, FASN overexpression reduced BLM-induced loss of mitochondrial membrane potential and the production of mitochondrial reactive oxygen species (ROS). Oleic acid, a fatty acid component increased by FASN overexpression, inhibited BLM-induced cell death in primary murine AECs and rescue BLM induced mouse lung injury/fibrosis. FASN transgenic mice exposed to BLM exhibited attenuated lung inflammation and collagen deposition compared to controls. Our findings suggest that defects in FASN production may be associated with the pathogenesis of IPF, especially mitochondrial dysfunction, and augmentation of FASN in the lung may have therapeutic potential in preventing lung fibrosis.

## Introduction

Idiopathic pulmonary fibrosis (IPF) is a disease involving chronic, progressive, irreversible interstitial pneumonia characterized by alveolar epithelial cell (AEC) injury with subsequent proliferation of activated fibroblasts known as myofibroblasts^1^. Although the molecular mechanism underlying the development of IPF remains unclear, repetitive excessive oxidative stress causing ACE damage has been suggested as the initial step of lung fibrosis followed by aberrant wound healing processes responsible for fibrotic remodeling^2^.

Type II AECs produce surfactant and contribute to repair of the lung following injury^3^. Pulmonary surfactant, a surface-active lipoprotein complex synthesized by type II AECs, is primarily composed of various types of saturated phospholipids and cholesterol along with lung-specific surfactant proteins^4^. Dysfunction of surfactant results in alveolar damage, and mutation of the surfactant protein C (SPC) gene causes familial pulmonary fibrosis^3,5^. There is accumulating evidence that the dysregulation of lipid metabolism within mitochondria of type II AECs may play a role in the development of IPF. For example, lipotoxicity from the accumulation of saturated fatty acids has been shown to play a role in the development of lung fibrosis in animal models. Sunaga et al. reported depletion of long-chain fatty acid family member 6 (Elovl6) in the lungs of patients with IPF and in BLM-treated mice^6^. Moreover, depletion of Elovl6 expression by small interfering RNA (siRNA) increases reactive oxygen species (ROS) and apoptosis, and thus promotes lung fibrosis. Similar results have been observed in a lung fibrosis model of mice deficient in stearoyl coenzyme A (CoA) desaturase-1, an enzyme that converts saturated fatty acids into monounsaturated fatty acids^7^.

Fatty acid synthase (FASN) is the principal enzyme responsible for the de novo synthesis of FAs^8^. FASN catalyzes the reductive synthesis of long-chain fatty acids from malonyl-CoA. Transcriptional profiling of IPF lungs has shown that FASN mRNA expression is decreased by 2.8-fold in IPF patients compared to controls^9^. In a murine model of BLM-induced lung fibrosis, FASN deficiency in type II AECs causes deterioration of fibrotic remodeling of the lung^10^. However, the precise role of FASN in AECs in the fibrotic process remains unclear.

This study demonstrated the beneficial effect of FASN on BLM-induced AEC death and mitochondrial dysfunction. Using FASN transgenic mice, we showed that FASN overexpression has a protective effect against BLM-induced lung inflammation and fibrosis. In addition, we discuss the possible underlying mechanisms.

## Results

### FASN expression is reduced in the lungs of IPF patients and BLM-treated mice

**Figure 1:**
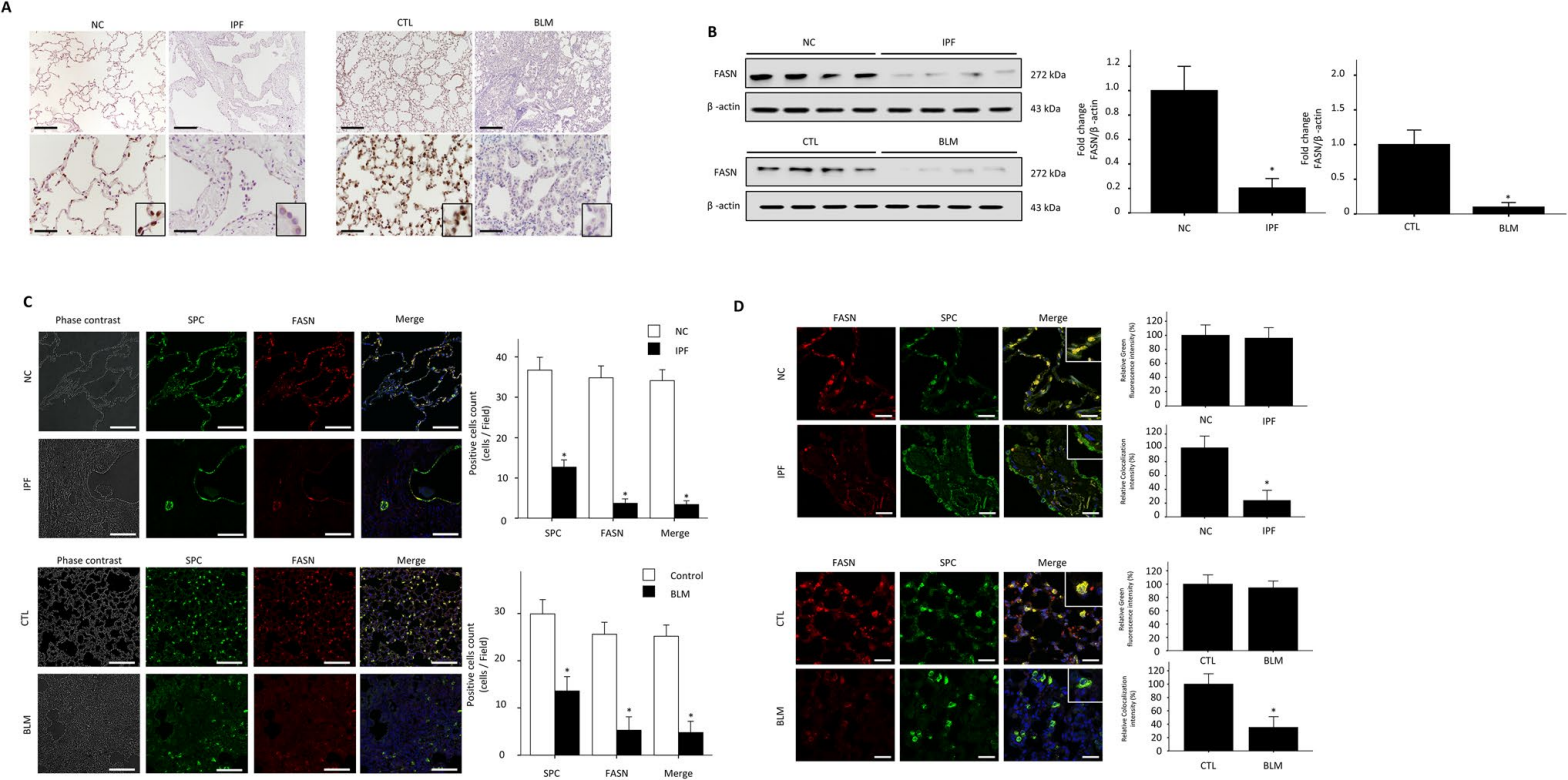
FASN protein levels were decreased in the lungs of patients with IPF and in BLM-treated mice. (**A**) Immunohistochemical staining of FASN in human and mouse lungs. FASN was strongly localized in AECs (inset) in NC or control mice. Original magnification: × 100, inset panels × 400. (**B**) Lung FASN expression was measured by immunoblotting. Densitometry of FASN bands. Equal amounts of protein obtained from lung lysates were subjected to immunoblotting with anti–FASN Ab. Cropped images are displayed, uncropped blots are displayed in Supplementary Fig. 5. The data are expressed as the mean ± standard error of the mean. The data were normalized to β-actin. *P < 0.05, vs. NC or CTL. (C) Historical analysis of combination of immunofluorescence staining with anti-FASN and anti–SPC in IPF and NC (upper panels, n = 6 per group), CTL and BLM (lower panels, n = 6 per group). SPC-positive cells (green), FASN cells (red), and DAPI (blue) are shown. (D) Representative immunofluorescence images showing FASN (red) in AECIIs (using anti‐SPC, green) of NC, IPF lungs (upper panels, n = 6 per group), CTL and BLM lungs (lower panels, n = 6 per group). Scale bar, 50 µm. The data are expressed as the mean ± standard error of the mean. *P < 0.05, vs. NC or CTL.

FASN is primarily expressed in AECs, and is not found in fibrotic areas in the IPF lung (Fig. 1A). Double-labeled immunofluorescence analysis showed that the majority of FASN–expressing cells were type II AECs (Fig. 1C). FASN expression was weaker in AECs near fibrotic areas in the IPF lung than in healthy controls. (Fig. 1A, D). FASN protein levels were significantly lower in the IPF group than in healthy controls (Fig. 1B). In the mouse lung, FASN was also primarily expressed in AECs (Fig. 1A, C), and the level of FASN expression was significantly diminished after 21 days of BLM exposure (Fig. 1B). In AECs isolated from BLM- treated mice, FASN expression also reduced compare than controls (Supplementary Fig. 1A). Similar to IPF, FASN expression was not observed in fibrotic areas of the mouse lung (Fig. 1A, C). To provide a clearer explanation for the decreased FASN protein levels in IPF and BLM exposed mice lungs, we quantified numbers of FASN-positive and SPC-positive cells and the intensities of FASN expressions in the AECs in the lungs. As expected, the numbers of AECs and FASN positive cells were dramatically decreased in IPF and BLM- treated lungs compared to controls (Fig. 1C). In addition, the expression intensities of FASN were significantly lower in areas with a similar number of AEC cells in IPF and BLM-treated lungs compared to controls. (Fig. 1D).

These findings indicate that FASN is constitutively expressed in AECs and that lower FASN protein levels in IPF and BLM treated lungs are due to both a reduced number of AEC cells in fibrotic areas and a decrease in FASN expression in AECs (Fig. 1C, D).

### Generation of FASN transgenic mice

**Figure 2:**
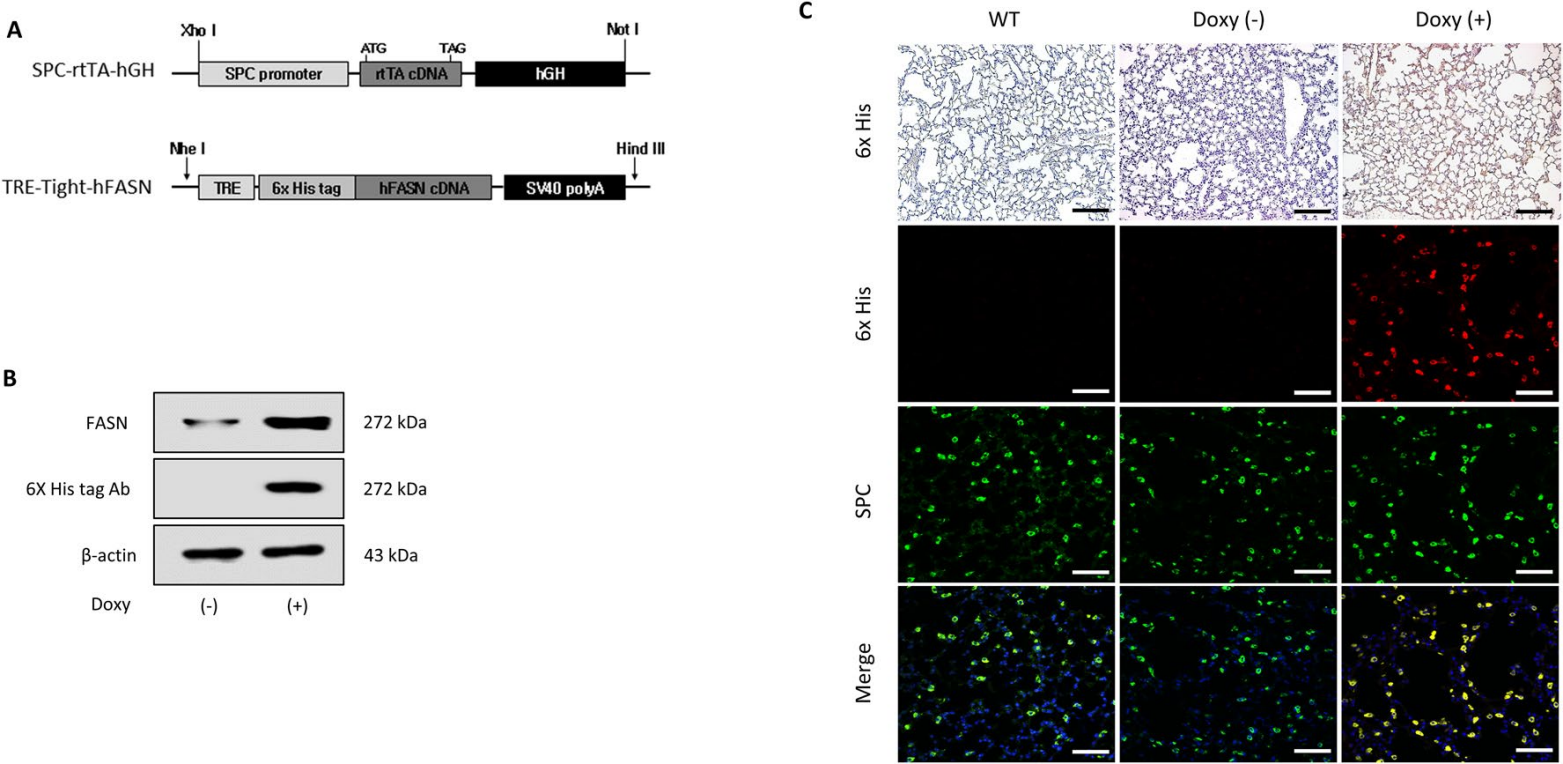
Generation of FASN transgenic mice. (**A**) Constructs for generation of FASN transgenic mice. (**B**) Immunoblotting analysis of human FASN- His in control and doxycycline-treated mice. FASN antibody detects both humans and mice FASN protein. Cropped images are displayed, uncropped blots are displayed in Supplementary Fig. 5. (**C**) Immunohistochemical stain of the lungs of wild type, control and doxycycline-treated FASN transgenic mice. Scale bar, 50 µm.

Transgenic mice harboring the human FASN gene in the C57BL/6/J background were generated as described previously^11^. Briefly, inducible human FASN transgenic mice were produced by coinjection of SP-C-rtTA-hGH and pTRETight-FASN into C57BL/6 blastocysts. In this system, the SPC promoter directs the expression of rtTA to the alveolar epithelium. In the presence of doxycycline, rtTA can bind in trans to tet-O, and the VP-16 transactivator activates human FASN gene transcription. In the absence of doxycycline, rtTA binding occurs at very low levels, and only low-level transcription of the gene was seen. The constructs for generation of FASN overexpressing transgenic mice are shown in Fig. 2A. Without treatment of doxy, FASN proteins levels were significantly decreased, but not absence due to FASN antibody detect both human and mouse FASN (Fig. 2B). Immunofluorescence stain showed that human FASN protein (6 × His tag, red) were colocalized with AECs (SPC, green) only after treatment with doxycycline (Fig. 2C).

### Effect of overexpression and knockdown of FASN on BLM-induced cell apoptosis and necrosis

**Figure 3:**
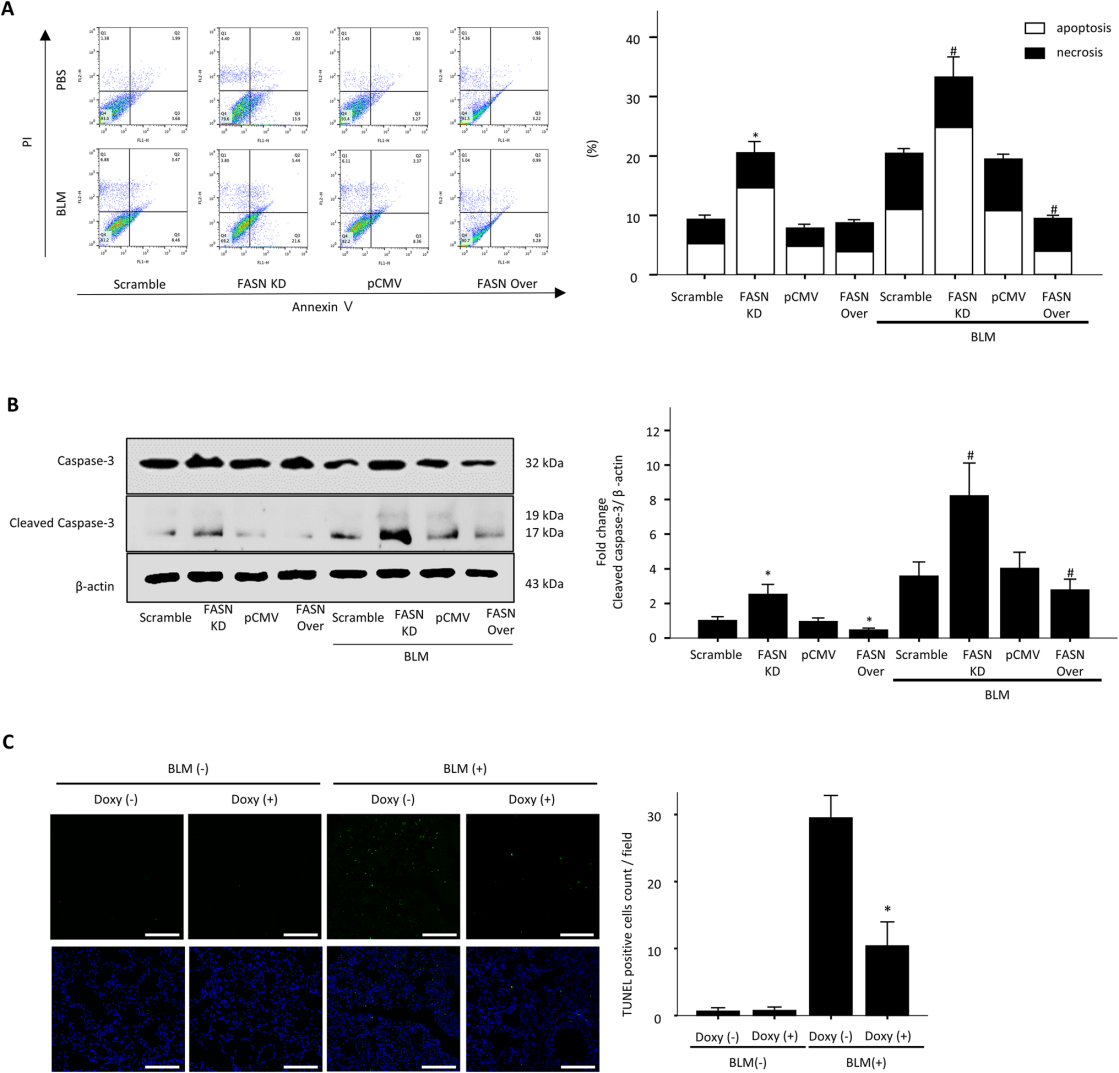
Effects of knockdown or overexpression of FASN on death of A549 alveolar epithelial cells and in the lungs of FASN transgenic mice. Stable FASN-overexpressing or FASN-knockdown cells were treated with BLM (10 μg/mL) for 24 h in serum-free medium. (**A**) Apoptosis was defined by annexin V+/both PI+ and PI− staining. Necrosis was defined by PI+/both Annexin V+ and Annexin V− staining. Quantification of the apoptotic and necrotic cell percentage. The data are expressed as the mean ± SEM (n = 4). *P < 0.05, vs. scramble or pCMV. # P < 0.05, vs. scramble + BLM or pCMV + BLM. (**B**) Protein expression of the total and active forms of caspase-3 was measured by immunoblotting. The intensity of the active form of caspase-3 was quantified by densitometry, and the data were normalized to β-actin. Cropped images are displayed, uncropped blots are displayed in Supplementary Fig. 5. The data are expressed as the mean ± SEM. Error bars represent mean ± SEM (n = 4) *P < 0.05, vs. scramble or pCMV. # P < 0.05, vs. scramble + BLM or pCMV + BLM. (**C**) Tissues stained by the TUNEL method were observed at × 100 magnification, and the number of TUNEL-positive cells in 20 fields per lung was counted (n = 8/group). The data are expressed as the mean ± SEM. * P < 0.05, vs doxy(−)/BLM (+).

Excessive oxidative stress-induced AEC damage is the main mechanism underlying the development and progression of lung fibrosis. As excessive oxidative stress cause A549 cells death by apoptosis or necrosis, we investigated the role of FASN in oxidative stress-induced cellular damage using A549 cells stably overexpressing His-tagged human FASN. We also performed lentiviral-mediated infection of A549 cells with human FASN shRNA (no. TL31058V; OriGene, Rockville, MD, USA), which was shown to efficiently knock down human FASN protein expression (supplementary Fig. 1B). Twenty-four hours after exposure of the cells to 10 μg/mL BLM, the proportions of apoptotic and necrotic cells were assessed by flow cytometry. FASN knockdown markedly increased the fractions of BLM-induced apoptotic and necrotic cells, both of which were significantly reduced by FASN overexpression (Fig. 3A). With BLM treatment, the levels of the cleaved form of caspase-3 protein were significantly decreased by FASN overexpression, but markedly increased by shRNA-mediated FASN knockdown (Fig. 3B). We also investigated whether BLM could induce apoptosis in mouse AECs. Our data reveal that the levels of the cleaved form of caspase-3 were increased in AECs isolated from mice treated with BLM (Supplementary Fig. 1A). To determine whether FASN attenuated BLM-induced increased apoptosis in vivo, we performed TUNEL assay in the mouse lungs. TUNEL assay revealed approximately 60% of fewer apoptotic cells in the lungs of FASN transgenic mice compared with the lungs of BLM-treated control mice (Fig. 3C). These results indicate that under conditions of cellular damage and death induced by BLM, FASN is essential for maintenance of cell survival, and overexpression of FASN can rescue cell death.

### FASN overexpression reduces BLM-induced loss of mitochondrial membrane potential and ROS production

**Figure 4:**
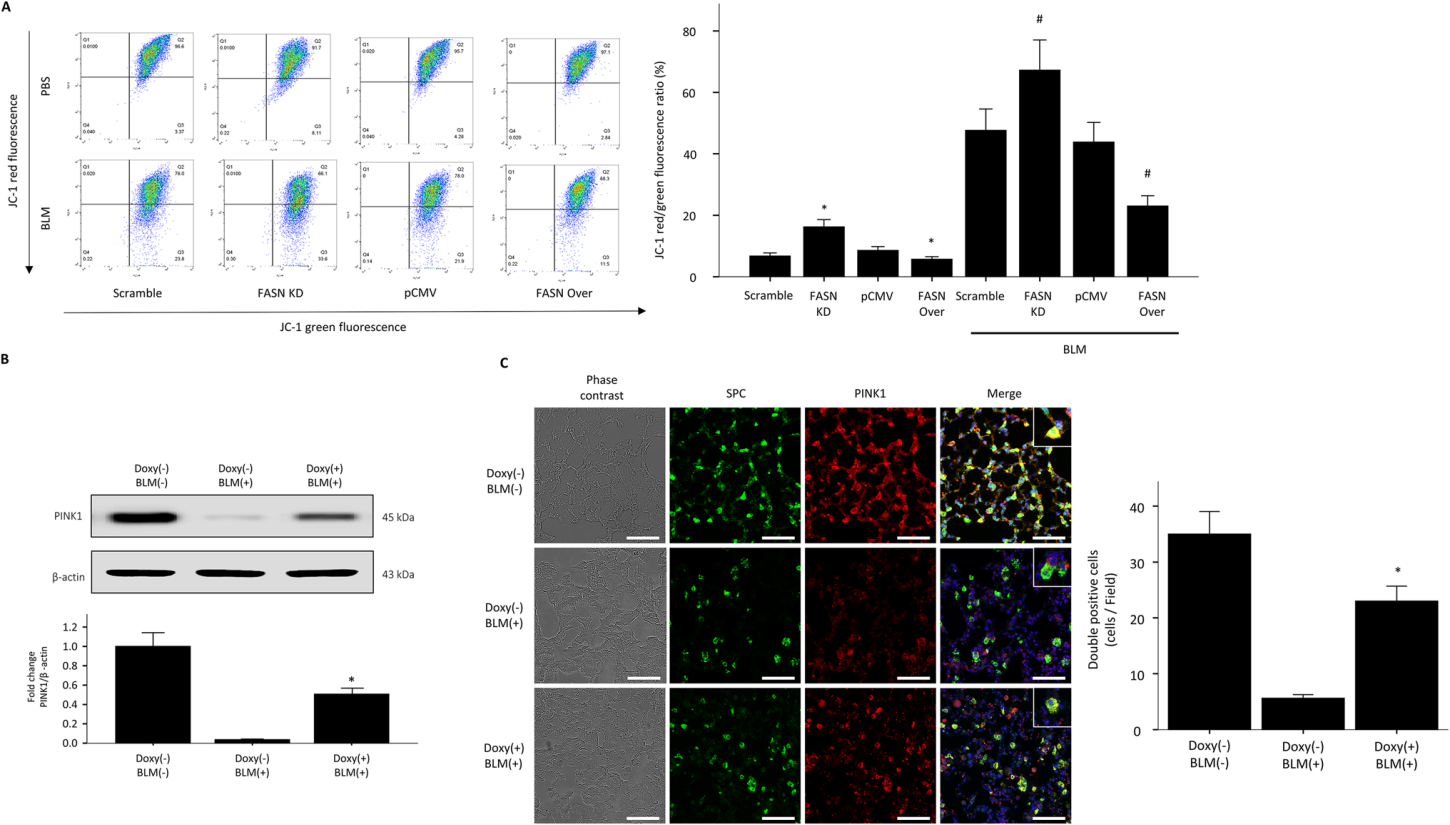
FASN regulates BLM-induced loss of mitochondrial membrane potential and mitochondrial dysfunction in AECs. FASN-overexpressing and knockdown cells treated with BLM (10 μg/mL) were stained with JC-1 for 30 min and analyzed by flow cytometry. (**A**) The proportion of depolarizing mitochondrial membrane potential (JC-1 green fluorescence) was increased in FASN knockdown cells, whereas it was decreased by FASN overexpression. Graph showing the ratio of the intensity of red to green fluorescence (JC-1 aggregate and JC-1 monomer) of flow cytometry. The data are expressed as the mean ± standard error of the mean (n = 4). *P < 0.05, vs Scramble or pCMV. #P < 0.05, vs Scramble + BLM or pCMV + BLM. (**B**) Protein expression of PINK1 was measured by Immunoblotting analysis in the lungs of FASN transgenic mice. The intensity of the PINK1 was quantified by densitometry, and the data were normalized to β-actin. Cropped images are displayed, uncropped blots are displayed in Supplementary Fig. 5. The data are expressed as the mean ± SEM. Error bars represent mean ± SEM (n = 6) *P < 0.05, vs. Doxy (−)/BLM (+). (**C**) Immunofluorescence analysis images of PINK1 (red) in AECIIs (SPC, green) of FASN transgenic mice along with the quantification of double positive cells (n = 6, each group). Scale bar, 50 µm. The data are expressed as the mean ± standard error of the mean. *P < 0.05, vs. Doxy (−)/BLM (−)panels × 400.

**Figure 5:**
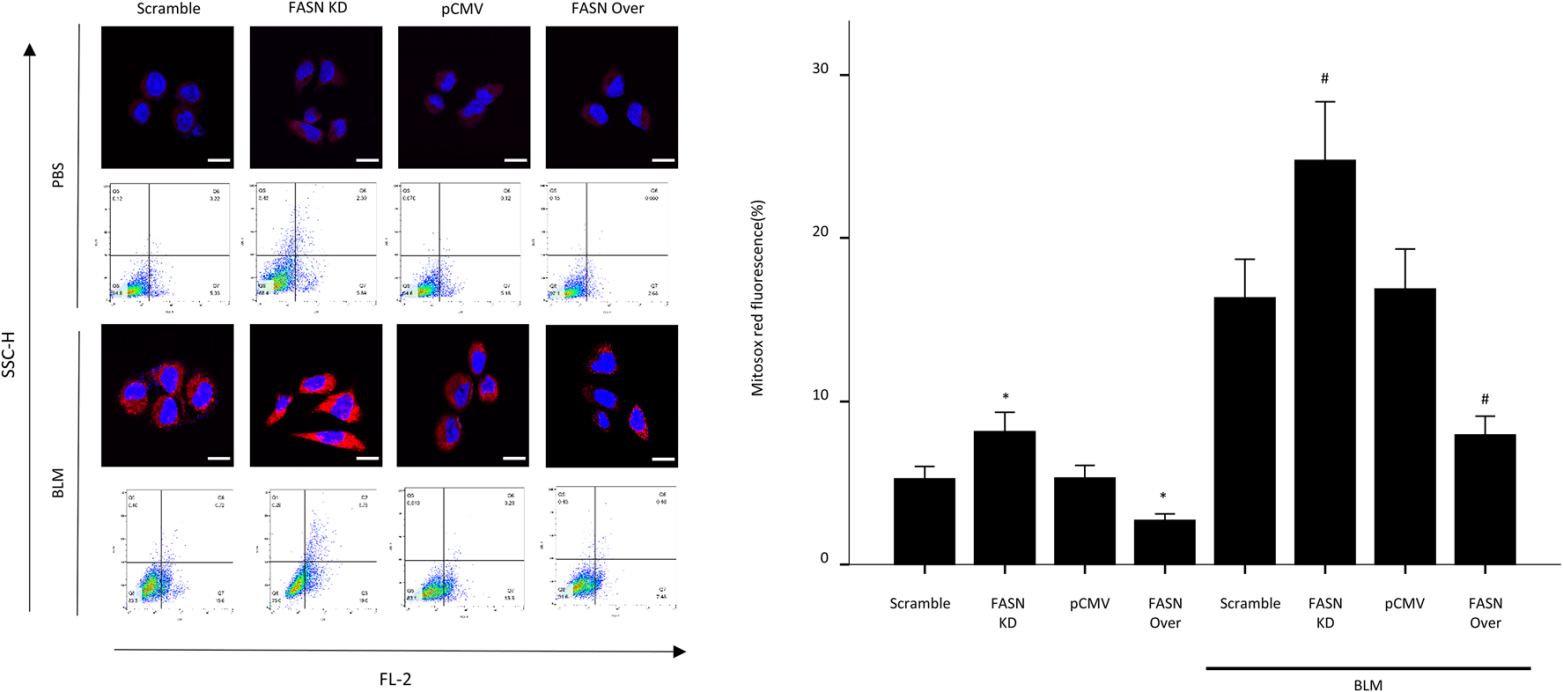
FASN regulates BLM-induced production of reactive oxygen species (ROS) in AECs. FASN overexpressing and knockdown cells were treated with BLM (10 μg/mL) for 24 h and analyzed by flow cytometry. Mitochondrial ROS production was detected with MitoSox-Red (red). The data are expressed as the mean ± standard error of the mean (n = 4). Scale bar, 50 µm. *P < 0.05, vs Scramble or pCMV. #P < 0.05, vs Scramble + BLM or pCMV + BLM.

The mitochondria are both the major sources of intracellular ROS generation and important targets for the damaging effects of ROS^12^. A recent study showed that FASN regulates mitochondrial apoptotic priming in cancer cells^13^. Therefore, we examined whether FASN may regulate mitochondrial function in vitro. Oxidative damage to the mitochondrial membrane causes depolarization of membrane potential^14^. In the basal state, the fluorescent probe JC-1 showed high mitochondrial potential in all cells (red fluorescence) and almost no membrane depolarization (green fluorescence) in controls. Membrane depolarization was significantly increased by FASN knockdown and downregulated by FASN overexpression (Fig. 4A). In the presence of BLM, FASN overexpression significantly suppressed mitochondrial membrane depolarization (Fig. 4A). In contrast, mitochondrial depolarization potentials were markedly increased by FASN knockdown (Fig. 4A). PINK1 is a key mediator of mitochondrial quality control and the level of PINK1 expression can serve as a marker of mitochondrial injury or dysfunction^15^. To evaluate whether overexpression of FASN rescue mitochondrial dysfunction in mice, lung PINK1 expressions were measured by western blotting and immunofluorescence. The western blot showed that lung PINK1 protein levels in BLM- treated FASN TG mice were significantly higher than those in control mice (Fig. 4B). Similarly, FASN overexpression increased BLM induced diminished in PINK1 expression in the AECs of the mice (Fig. 4C). These data suggest that decreased FASN levels result in increased susceptibility to mitochondrial damage, and overexpression of FASN has a protective effect against oxidative stress-induced mitochondrial damage in AECs both in vitro and in vivo. Next, we measured mitochondrial superoxide generation using MitoSox-Red as a probe in FASN overexpressing/knockdown cells. Under both basal conditions and in the presence of BLM, FASN knockdown significantly increased mitochondrial superoxide level compare to controls (Fig. 5). FASN overexpressing cells showed markedly reduced mitochondrial ROS production compared to control or FASN knockdown cells (Fig. 5). Taken together, our observations suggest that FASN is important for maintenance of mitochondrial function against BLM-induced oxidative stress, and overexpression of FASN can alleviate oxidative stress-induced mitochondrial dysfunction.

### Overexpression of FASN increases oleic acid production and oleic acid diminishes BLM-induced AEC death, and attenuate BLM-induced lung injury/fibrosis

**Figure 6:**
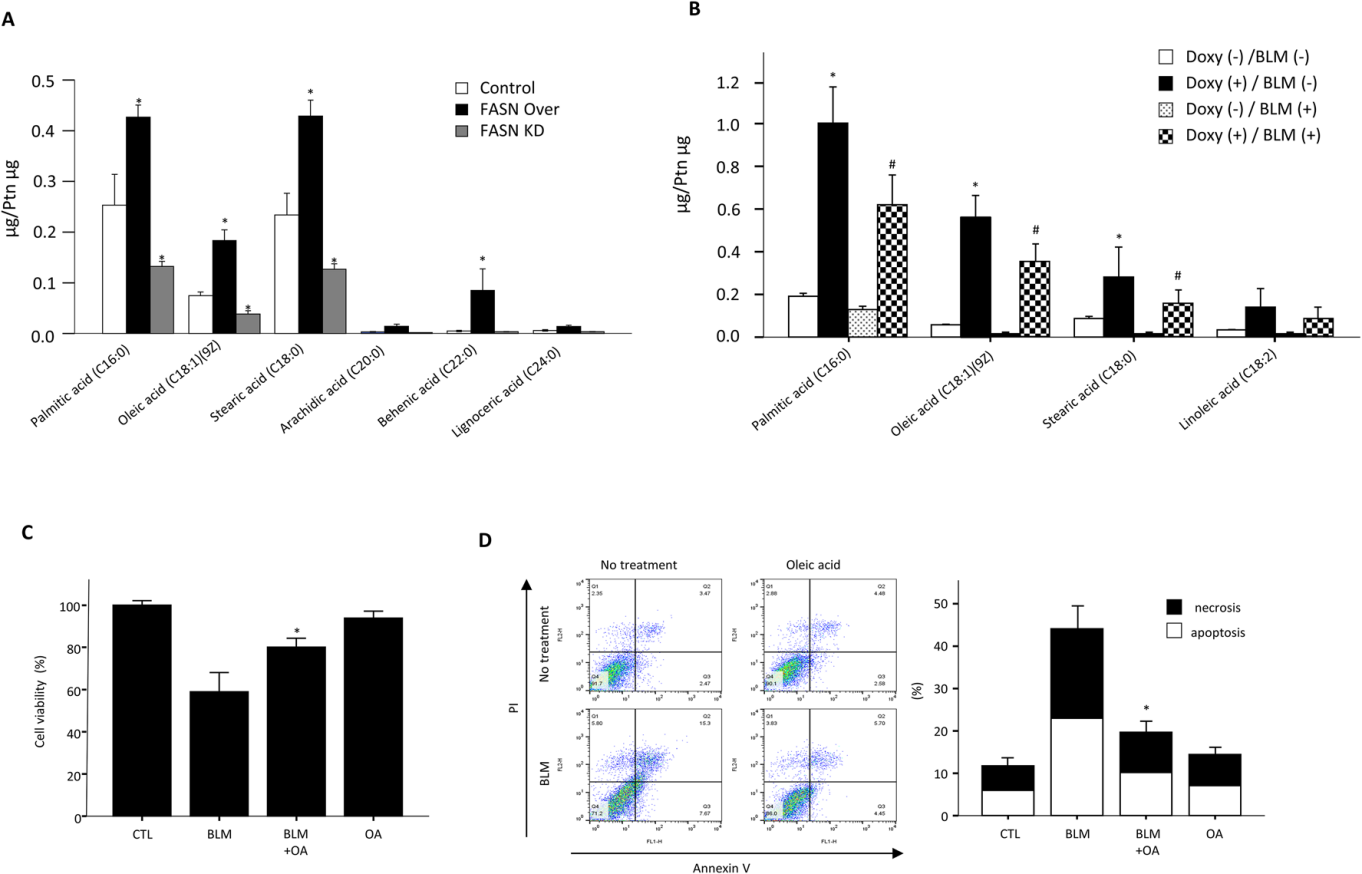
Fatty acid profiles of FASN overexpressing and knockdown cells and AECs from FASN transgenic mice, and beneficial effect of oleic acid on BLM -treated AECs. (**A**) Changes in fatty acid composition by overexpression or knockdown of the cells were determined by LC–MS. (**B**) Changes in fatty acid composition in AECs from FASN transgenic mice lung were determined by LC–MS. * < 0.05, Doxy(−)/BLM (−) vs Doxy (+)/BLM (−). #P < 0.05, Doxy (−)/BLM (+) vs Doxy (+)/BLM (+). (**C**) The WST-1 assay showed that treatment with oleic acid (100 μM) improved survival of BLM (10 μg/mL)-treated primary mouse AECs. (**D**) Treatment with oleic acid (100 nM) rescued AECs from BLM (10 μg/mL)-induced apoptotic cell death. Apoptosis was defined by Annexin V+/both PI+ and PI− staining. Necrosis was defined by PI+/both Annexin V+ and Annexin V− staining. The data are expressed as the mean ± standard error of the mean (n = 4). * < 0.05, BLM vs BLM + OA.

The main function of FASN is to catalyze the de novo biosynthesis of various long-chain saturated fatty acids, including palmitic acid. We analyzed the fatty acid composition of FASN-overexpressing and knockdown cells, as well as AECs from FASN transgenic mice, using LC–MS. Palmitic, oleic, and stearic acids were produced in the cells. As expected, overexpression of FASN significantly increased palmitic acid level, which was markedly reduced by FASN knockdown compared to controls (Fig. 6A). The levels of oleic acid and stearic acid were also significantly increased in FASN-overexpressing cells and decreased by FASN knockdown (Fig. 6A). Similarly, we found increased production of palmitic acid, stearic acid, and oleic acid in the AECs from FASN transgenic mice (Fig. 6B). Additionally, these lipid components remained elevated even after BLM exposure (Fig. 6B). To determine whether the beneficial activities of FASN overexpression are related to changes in fatty acid compositions, we examined the effects of oleic acid on BLM-treated primary murine AECs. Cell survival was increased by oleic acid in BLM-treated AECs (Fig. 6C). The BLM-induced increases in apoptotic and necrotic cell death were markedly reduced by oleic acid (Fig. 6D). However, treatment with palmitic acid and stearic acid did not reduce cell death in BLM -exposed AECs (Supplementary Fig. 2). Next, we examined whether oleic acid has a beneficial effect on the BLM -induced lung injury/fibrosis model. After intratracheal injection of BLM, oleic acid (3 mg/kg) was administered immediately by oral gavage every 2 days, and the mice were sacrificed after 21 days. H&E stain showed that treatment with oleic acid attenuated BLM- induced lung inflammation and structural distortion (Supplementary Fig. 3A). BALF analysis showed that oleic acid significantly reduced lung inflammatory cell numbers (Supplementary Fig. 3B). Furthermore, oleic acid reduced lung fibrosis caused by collagen deposition, as shown by Masson trichrome staining and hydroxyproline assay (Supplementary Fig. 3C, D). These observations suggest that the beneficial effects of FASN maybe at least partly attributable to the increased production of oleic acid in vivo.

### Overexpression of FASN prevents BLM-induced lung inflammation and fibrosis

**Figure 7:**
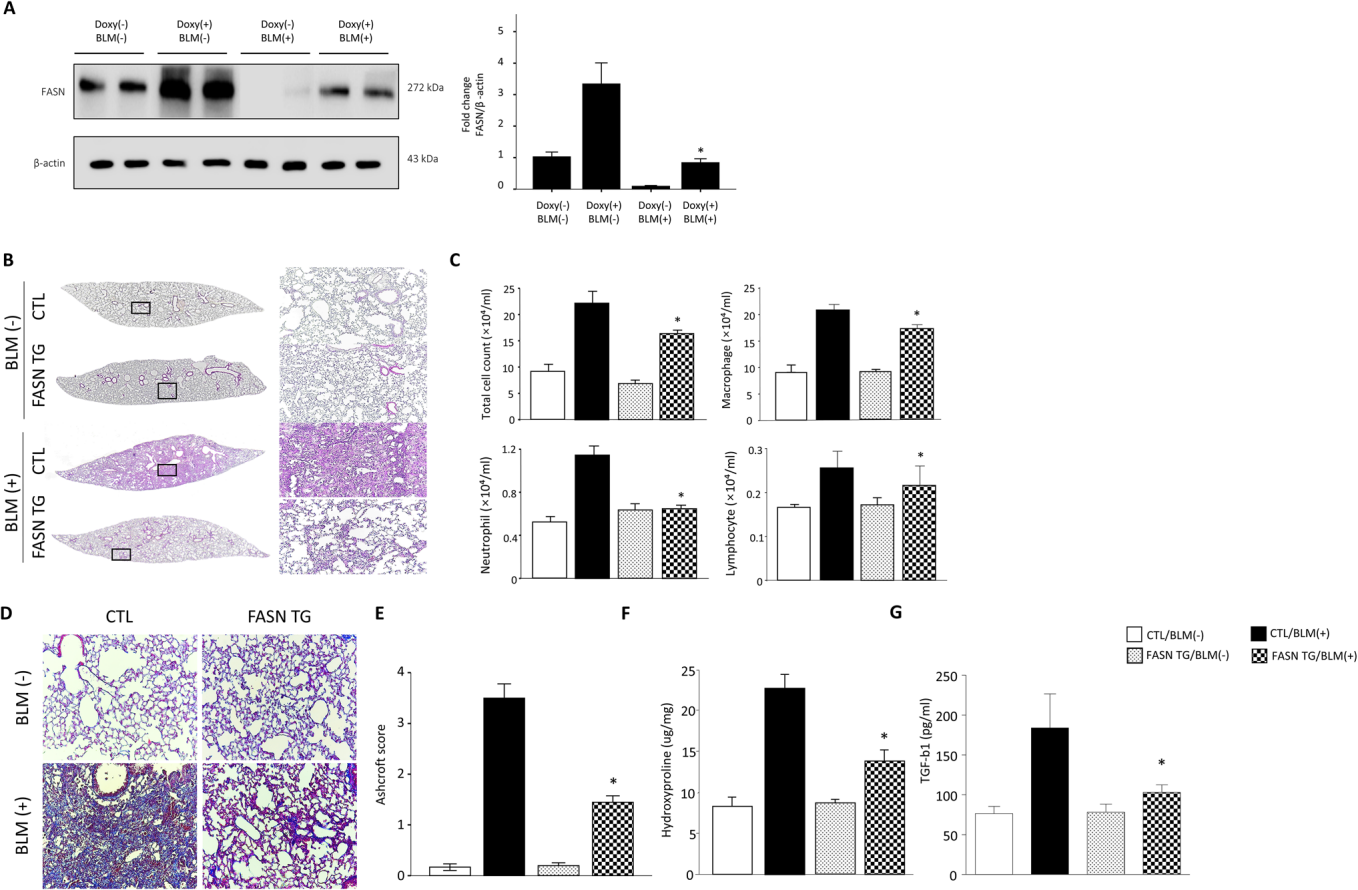
FASN overexpression attenuates BLM- induced lung inflammation and fibrosis in a mouse model. (**A**) Protein expression of FASN was measured by immunoblotting. The intensity of FASN was quantified by densitometry, and the data were normalized to β-actin. Cropped images are displayed, uncropped blots are displayed in Supplementary Fig. 5. The data are expressed as the mean ± SEM. Error bars represent mean ± SEM (n = 6) *P < 0.05, vs. Doxy (−)/BLM (+). (**B**) H&E staining of control and BLM-treated FASN transgenic mouse lungs. Original magnification × 10; original magnification × 100 (**C**) Cell counts in BALF, collected on day 21. The total number of cells was counted using a hemocytometer. Differential cell counts in BALF were analyzed from 500 cells stained with Diff-Quick (n = 8 per group). (**D**) Masson’s trichrome staining in control and BLM-treated FASN transgenic mouse lungs. Original magnification × 200. (**E**) Quantification of lung fibrosis by Ashcroft score (n = 8 per group). Data are expressed as the mean ± SEM. (**F**) Collagen measurement by the hydroxyproline assay of control and BLM-treated mouse lungs (n = 8 per group). (**G**) Active TGF-β1 levels in control and BLM-treated mouse lungs (n = 8 per group). TGF-β1 levels in lung lysates were measured by ELISA. *P < 0.05 CTL/BLM(+) vs. FASN TG/BLM(+).

Next, we examined whether overexpression of FASN could prevent fibrotic activity in the BLM-induced lung fibrosis model. FASN transgenic mice were treated with doxycycline and BLM on Day 0 and lung samples were evaluated on day 21. Immunoblot analysis showed that the FASN protein levels were still highly expressed in the lungs of BLM-treated FASN overexpression mice compared to control lungs (Fig. 7A). Histochemical staining with hematoxylin and eosin (H&E) showed that FASN overexpression efficiently ameliorated BLM-induced lung structural distortion (Fig. 7B). Consistent with the results of H&E staining, BALF analysis showed that FASN overexpression significantly attenuated lung inflammation and reduced the total numbers of macrophages, neutrophils, and lymphocytes (Fig. 7C). Furthermore, overexpression of FASN considerably reduced lung fibrosis caused by collagen deposition, as shown by Masson’s trichrome staining and Ashcroft scoring (Fig. 7D, E). The hydroxyproline assay further confirmed the antifibrotic activity of FASN overexpression during BLM treatment (Fig. 7F). The level of the active form of TGF-β1 was significantly increased in the lungs following exposure to BLM but this was significantly attenuated by overexpression of FASN (Fig. 7G).

### Effect of doxycycline on BLM-induced lung inflammation and fibrosis in mouse lung

Doxycycline reportedly has an anti-fibrotic effect on BLM-induced experimental fibrosis^16^. To determine whether treatment with doxycycline contributed to the anti-fibrotic effect on FASN transgenic mice, BLM was administered intratracheally to the C57BL/6J mice (8 weeks, Male) with or without doxycycline-containing water. The histologic findings revealed significant accumulation of inflammatory cells and interstitial edema in both the BLM-exposed mice receiving doxycycline and those receiving drinking water without doxycycline (Supplementary Fig. 4A). The total numbers of inflammatory cells, macrophages and neutrophils in the BAL fluid tended to decrease, but not significantly, in the group that received doxycycline compared to the group that not receive doxycycline (Supplementary Fig. 4B). There was no significant differences in lung fibrosis between the group that received doxycycline and the group that did not (Supplementary Fig. 4C, D). Therefore, our data suggest that doxycycline, per se did not significant affect lung fibrosis induced by BLM in mice.

## Discussion

We found that FASN levels were markedly decreased in the lungs of patients with IPF as well as BLM-exposed mice. Overexpression of FASN reduced BLM-induced apoptotic cell death, which are major component of the pathophysiology of IPF. Our findings indicate that overexpression of FASN protects against BLM-induced lung inflammation and fibrosis, and these beneficial effects are associated with stabilized mitochondrial membrane potential and reduced mitochondrial ROS production. This is the first report of the protective effect of FASN overexpression against BLM-induced lung injury/fibrosis along with a discussion of the possible underlying mechanism.

FASN is an integral anabolic enzyme in the de novo synthesis of fatty acids. It catalyzes the synthesis of long-chain fatty acids from malonyl-CoA. Its role has been investigated based on recognition of the critical interrelation of metabolic dysregulation and the progression of various types of tumors. FASN levels are significantly increased in numerous human tumors, including breast^17^,—prostate^18^, and colorectal cancers^19^. However, the role of FASN in the pathogenesis of IPF has not been studied to date.

The apoptotic death of ACEs by oxidative stress has been implicated in the pathogenesis of IPF^2^. Our results show that FASN overexpression significantly reduces the increase in BLM-induced apoptotic cell death, while FASN knockdown results in a marked increase (Fig. 4A). Our data are in line with a recent study that showed that inhibition of FASN could trigger cancer cell death via interactions with the BCL-2 family and that upregulation of pro-death BH3 only proteins increase mitochondrial apoptosis priming^13^. Another recent study showed that FASN overexpression ameliorates genotoxic insult induced cancer cell death by upregulating poly (ADP-ribose) polymerase 1 and DNA repair via NF-κB and SP1^20^. Our data also confirm that FASN is crucial for the maintenance of the survival of ACEs against BLM induced cellular damage and that overexpression of FASN can rescue cells from death.

Mitochondria are involved in the synthesis of fatty acids, and proper mitochondrial function is required for lipid metabolism. Bueno et al. reported that FASN deficit causes metabolic dysregulation, especially impaired glycolysis and anaplerotic shift of the Krebs cycle in the mitochondria^21^. Therefore, we investigated whether changes in FASN expression in AECs affect mitochondrial functions, especially under conditions of oxidative stress. Our data show that FASN overexpression protects mitochondrial membrane potential against oxidative stress and attenuates mitochondrial ROS production, whereas knockdown of FASN aggravates mitochondrial dysfunction. Furthermore, we confirmed that FASN overexpression improved mitochondrial dysfunction in AECs of mice as shown increased expression of PINK1, a marker of mitochondrial dysfunction (Fig. 4C). Our FASN knockdown data are similar to those of a previous study that found that FASN deficiency of type II AECs results in impaired mitochondrial function under oxidative stress in mice^22^. However, there have been no reports that increasing expression of FASN may regulate mitochondrial function. Here, we showed that overexpression of FASN in AECs rescued mitochondrial dysfunction induced by BLM (Fig. 4). There is accumulating evidence that mitochondrial dysfunction contributes to the development and progression of chronic lung diseases^23^. Recently, decreased mitochondrial mass with morphological alteration and increased mitochondrial membrane depolarization characteristic features of mitochondrial dysfunction were observed in patients with IPF lungs^24,25^. Therefore, our data provide insights into the potential of FASN as a therapeutic target of mitochondrial dysfunction in IPF.

Transcriptomic analysis showed that FASN mRNA levels were significantly decreased in the IPF lung compared to healthy controls^26^. Here, we confirmed that FASN protein levels were decreased in the lungs of human patients with IPF and in BLM-induced fibrosis mice. In addition, FASN was mainly expressed in AECs, and BLM exposure decreased FASN expression in these cells. (Fig. 1 and Supplementary Fig. 1). In the IPF lung, its expression was weaker in AECs near the fibrotic areas than in healthy controls (Fig. 1A). Moreover, its expression was not found in the fibrotic areas of either group. These observations correspond with a previous report that FASN is expressed in type II AECs but not in fibroblasts^27^. Taken together, our data indicate that the reduced FASN levels in IPF or BLM- exposed lungs can be explained by the decreased numbers of AECs in the fibrotic area and weak expression of FASN in AECs (Fig. 1). Beneke et al.^27^ showed that increased FASN expression is critical for the biosynthesis of lung surfactant. Fatty acids are integral components of pulmonary surfactant, a mixture of phospholipids and specific proteins that lines the alveolar surface and is necessary for normal lung function. There is accumulating evidence that surfactant dysfunction occurs during IPF progression or exacerbation^28–30^. Therefore, we speculated that decreased FASN expression in IPF lungs may be linked with surfactant dysfunction. However, further studies are needed to evaluate the contribution of this interaction in lung fibrosis.

We showed that FASN expression was decreased in the IPF lung, and that mitochondrial dysfunction was attenuated by FASN overexpression in vitro. These findings support the potential benefits of FASN in vivo. To address this issue, we examined the efficacy of FASN overexpression in the BLM-treated lung fibrosis model. As shown in Fig. 7, overexpression of human FASN in AECs markedly attenuated BLM-induced lung fibrosis. These results are different from those of Jung et al.^31^, who examined the inhibitory effect of FASN in a BLM-induced lung fibrosis model. They reported that FASN inhibition decreased BLM-induced lung fibrosis and that FASN was required for TGF-β1 signaling^31^. Moreover, in contrast to our data, treatment of BLM increased FASN protein expression in the mouse lung in their study^31^. However, as shown in Fig. 1, both immunohistochemical and Western blotting analyses showed that FASN protein level were significantly decreased in both IPF lungs and BLM treated murine lungs. Our data are more compelling than those of Jung et al., as we examined FASN expression in IPF lung tissues by immunohistochemical and Western blotting analyses. Moreover, a previous study that used microarray analysis of data from another cohort also showed that FASN mRNA levels were significantly decreased in IPF^26^.

As FASN is crucial for the de novo biosynthesis of long-chain fatty acids^8^, we examined whether overexpression or knockdown of FASN regulated long-chain fatty acid production in vitro. As shown in Fig. 6A, overexpression and knockdown of FASN significantly increased or decreased the production of long-chain fatty acids, such as palmitic acid, oleic acid, and stearic acid, respectively. Similarly, the levels of palmitic acid, stearic acid and oleic acids were increased in the AECs of FASN overexpressing mice (Fig. 6B). These data are important for two reasons. First, as they demonstrate that FASN knockdown is mediated by FASN-specific activity and not unknown/off-target effects. Second, the overexpression of human FASN protein is functionally intact in mouse AECs. Next, we investigated the impacts of these long chain fatty acids on cell proliferation and survival in primary cultured AECs. Treatment with oleic acid had a beneficial effect on BLM-induced AEC death (Fig. 6D). These findings are consistent with previous data showing that oleic acid show cytoprotective activity in various cells by upregulating lipid peroxidation mediators^32,33^. In contrast, oleic acid induces apoptosis and cell death in cancer and neuronal cells^34,35^. Our data suggest that oleic acid has antiapoptotic activity and can rescue cell death in AECs by exposure to BLM. Additionally, orally administrated oleic acid attenuate BLM induced lung inflammation and fibrosis. These findings help explain, at least in part, beneficial effect of FASN overexpression on BLM induced lung injury/fibrosis.

This study had some limitations. We generated FASN-overexpressing and knockdown cell lines from A549 cells, a human lung cancer cell line. As lipid metabolism differs between cancer cells and normal cells, the effects of FASN on oxidative effects in the mitochondria may not be the same in A549 cells and normal cells. In addition, our findings do not explain the precise mechanisms underlying the beneficial effect of FASN overexpression on mitochondrial dysfunction. We assumed that excessive free fatty acids produced by FASN overexpression were broken down to acetyl-CoA, which then enhanced mitochondrial respiration and upregulated mitochondrial biogenesis through fatty acid oxidation. However, further studies are needed to evaluate this hypothesis.

Although many parts of the mechanism involved in the development and progression of IPF have been elucidated, a better understanding of the fibrogenic process is required to improve treatment options for this disease. The present study demonstrates decreased FASN expression in AECs in IPF and shows that FASN inhibition induces increases in cell death and mitochondrial dysfunction in vitro, suggesting that overexpression of FASN in vivo may provide an effective target for treatment of IPF.

## Materials and methods

### Reagents and antibodies

The antibodies (Abs) used rabbit anti-FASN polyclonal antibodies (no. ab22759; 1:500; Abcam, Cambridge, UK), anti-β-actin (no. A1978; 1:5000; Sigma-Aldrich, St. Louis, MO, USA), anti-prosurfactant protein C (no. ab90716; Abcam, Cambridge, UK), mouse anti-6X His tag (no. ab77825; 1:500; Abcam), anti-caspase-3 (no. 9662s; 1:500; Cell Signaling Technology, Danvers, MA, USA), anti-cleaved caspase-3 (no. 9661s; 1:500; Cell Signaling Technology, Danvers, MA, USA), PINK1 (no. 23274-1-AP; 1:500; proteintech; Suite 400 Rosemont, USA) with horseradish peroxidase-conjugated anti-rabbit or anti-mouse immunoglobulin G (IgG) secondary antibodies (no. SA001-500, SA002-500; GenDEPOT, Katy, TX, USA). Bleomycin hydrochloride (BLM; Nippon Kayaku, Tokyo, Japan), the annexin V-fluorescein isothiocyanate (FITC)/propidium iodide detection kit (BD Biosciences Pharmingen, San Diego, CA, USA), the JC-1 Assay Kit (no. M34152; Invitrogen, Paisley, UK), fluorescent MitoSox-Red reagent (no. M36008; Invitrogen), Fluoroshield mounting medium with DAPI (no. ab104139; Abcam), oleic acid (no. O1008; Sigma-Aldrich), the water-soluble tetrazolium salt (WST)-1 assay (no. 5015944001; Roche Diagnostics, Basel, Switzerland), SP-C-rtTA-hGH (courtesy of Dr. Jeffery Whitsett, Cincinnati Children’s Hospital Medical Center, Cincinnati, OH, USA), Masson’s trichrome staining kit (no. KTMTR; American Mastertech, Lodi, CA, USA), the hydroxyproline assay (no. MAK008; Sigma-Aldrich), the mouse TGF-β1 ELISA kit (no. DY1679; R&D Systems, Minneapolis, MN, USA), and the cell death detection kit (no. 11684795910; Roche) were obtained from the sources shown.

### Human lung samples

All human lung tissue samples from patients with IPF (n = 6) and control subjects (n = 6) were obtained from the Biobank at Soonchunhyang University Bucheon Hospital (Bucheon-si, South Korea). Histological diagnoses of usual interstitial pneumonia were confirmed based on surgical lung biopsy specimens. The control samples were normal, nondiseased lung tissue specimens obtained from patients who had undergone surgery for lung cancer. The study protocol was approved by the local ethics committee of Soonchunhyang University Bucheon Hospital (SCHBC_IRB_2016–12–024–002). Informed written consents for study participation and sample donation were obtained from the subjects. All procedures were performed in accordance with the Declaration of Helsinki of the World Medical Association.

### Immunohistochemical staining

Lung tissues were dehydrated and embedded in paraffin. For histological examination, sections 4 µm thick and bronchoalveolar lavage fluid (BALF) cells on slides were treated with 1.4% H_2_O_2_–methanol for 30 min to block endogenous peroxidase. Then, nonspecific binding was blocked with 1.5% normal serum, and slides were incubated with rabbit anti-FASN polyclonal antibodies (1:200; Abcam, Cambridge, UK), mouse anti-6X His tag monoclonal antibody (1:200; Abcam). The next day, the sections were incubated with ABC kit reagents (Vector Laboratories, Burlingame, CA, USA). The color reaction was developed by staining with a liquid 3,3′-diaminobenzidine positive-substrate kit (Golden Bridge International, Inc., Mukilteo, WA, USA). After immunohistochemical staining, the slides were counterstained with Harris’s hematoxylin for 1 min.

### Mouse AEC isolation and culture

Primary mouse AECs were isolated from wild-type mice using a previously described protocol with minor modifications^36,37^.

### Preparation of lung cell suspensions

Crude cell suspensions were prepared from C57BL/6 mice. The lungs were perfused with 0.9% NaCl using a 10 mL syringe fitted with a 21-gauge needle (BD Pharmingen, San Diego CA, USA) through the right ventricle of the heart until they were visually free of blood. A 21-gauge intravenous catheter was inserted into the trachea and secured with a suture. The lungs were filled with 1–2 mL dispase via the tracheal catheter and then allowed to collapse naturally, expelling part of the dispase. Low-melting point agarose (l%, 0.45 mL, stored in a 45 °C water bath; Invitrogen) was slowly infused via the catheter. The lungs were immediately covered with crushed ice and incubated for 2 min. Then they were removed and placed in 12 mL polypropylene culture tubes with 2 mL dispase (Sigma-Aldrich), incubated for 45 min at room temperature, and kept on ice until the next step. The lungs were transferred to 7 mL Dulbecco’s modified Eagle’s medium (DMEM) with 0.01% DNase I in 60 mm Petri dishes. The digested tissue was carefully teased from the airways with the curved edge of fine-tipped forceps and gently swirled for 5–10 min. The resulting suspension was successively filtered through 100 µm and 40 µm Falcon cell strainers and then through 25 µm nylon mesh. The filtered suspension was centrifuged at 130 × g for 8 min at 4 °C and resuspended in 10 mL 10% fetal bovine serum (FBS) and 1% penicillin–streptomycin in 25 mM 4-(2-hydroxyethyl)-1-piperazineethanesulfonic acid (HEPES)-buffered DMEM.

### Magnetic purification of type II AECs from crude cell suspensions

The cells were incubated with biotinylated anti-CD32 (0.5 µg/million cells; BD Pharmingen) and biotinylated anti-CD45 (1.5 µg/10^6^ cells; BD Pharmingen) for 30 min at 37 °C. Streptavidin-coated magnetic particles (Thermo Fisher Scientific, Waltham, MA, USA) were washed twice in phosphate-buffered saline (PBS) (10 min per wash) in polypropylene culture tubes using a magnetic tube separator (Sigma-Aldrich). After incubation, the cells were centrifuged (130 × g for 8 min at 4 °C), resuspended in 7 mL DMEM, added to the magnetic particles, and incubated with gentle rocking for 30 min at room temperature. At the end of incubation, the tubes were attached to the magnetic tube separator with adhesive tape for 15 min. The cell suspension was aspirated from the bottom of the tube using a narrow-stemmed transfer pipette, centrifuged, and resuspended in culture medium. Cell viability was determined by trypan blue staining. The purity of type II AECs was assessed by pro-SPC immunofluorescence staining. As in previous studies, 4–8 × 10^5^ cells were isolated from a single mouse. In these samples, the purity of type II pneumocytes was 90%–93%. Isolated cells were maintained in 10% FBS and 1% penicillin–streptomycin in 25 mM HEPES-buffered DMEM.

### Cell culture

Human lung epithelial A549 cells (ATCC, Manassas, VA, USA) were cultured in DMEM or RPMI medium supplemented with 10% FBS (Thermo Fisher Scientific), 100 U/mL penicillin, and 100 μg/mL streptomycin (Gibco, Carlsbad, CA, USA). The cells were maintained in a humidified atmosphere with 5% CO2 at 37 °C.

### Generation of human FASN constructs

Human FASN cDNA (no. HG12314-NH; Sino Biological, Beijing, China) was ligated into pCMV3-N-His tag plasmid digested with *Kpn*I and *Xba*I using T4 ligase at an insert to vector molar ratio of 3:1. The ligation mixture was transformed into competent Escherichia coli JM109 (Promega, Madison, WI, USA), and plated on LB-amp plates. After selection of positive colonies, they were incubated with LB broth in a shaking incubator at 37 °C for 24 h. Plasmid DNA was extracted from LB broth using a QIAGEN Plasmid mini prep kit (QIAGEN, Venlo, The Netherlands) according to the manufacturer’s protocol.

### Generation of cell line with stable expression of human FASN-His

A549 cells were seeded in 6-well plates and grown in antibiotic-free medium until 70–80% confluence. The pCMV FASN-His tag plasmid was transfected into the cells using Lipofectamine 2000 (Invitrogen) in accordance with the manufacturer’s recommendations. Briefly, solution A contained 250 µL Opti-MEM (Gibco) and 4 µg plasmid DNA. Solution B contained 250 µL Opti-MEM and 10 µL Lipofectamine 2000. The two solutions were gently mixed and incubated at room temperature for 20 min. Lipid–DNA complexes were overlaid onto the cells and incubated at 37 °C for 48 h in a tissue culture incubator under 5% CO2. Then the medium was changed to fresh medium containing 1000 µg/mL G418 and further incubated. G418-resistant clones were isolated after 192 h, and individual clones were transferred into the wells of 24-well plates. The medium was changed to fresh medium supplemented with antibiotic 1 day later. When cells grew to 70% confluence, they were subcultured and expanded by continuous transfer from 6-well plates to fresh 6 cm plates and then to 10 cm plates. Overexpression of the FASN-His tag was confirmed by Western blotting analysis.

### Lentiviral transduction for FASN depletion in A549 cells

Transient and stable transfection procedures were performed with small hairpin (sh) RNA Lentiviral Transduction Particles according to the manufacturer’s instructions (Sigma-Aldrich). The shRNA against the FASN gene (FASN shRNA) and the corresponding control shRNA (Sigma-Aldrich) were used for RNA interference. For lentiviral transduction, A549 cells were cultured at 37 °C under 5% CO_2_ in 100 mm culture dishes containing 10% FBS (Gibco), 100 units/mL penicillin, 100 mg/mL streptomycin (Gibco), and 10 μL/mL  l-glutamine (Gibco). When cells reached 70% confluence, transduction was performed according to the manufacturer’s instructions (Sigma-Aldrich). Then, human-FASN shRNA-expressing lentivirus (FASN knockdown) or nontargeting shRNA-expressing scrambled RNA sequence (scramble) was added to the cells at a multiplicity of infection (MOI) of 5. The medium was changed after 24 h. Three days after transduction, stable clones expressing FASN shRNA and scramble RNA were selected over a further 10 days using 5 μg/mL puromycin (Sigma-Aldrich). The medium was changed every 48 h. Gene silencing was confirmed by Western blotting and reverse transcription polymerase chain reaction (RT-PCR).

### Western blotting

Proteins were extracted from lung tissues or cells in lysis buffer (Thermo Fisher Scientific) using protease and phosphatase inhibitor cocktail (Roche Diagnostics), followed by centrifugation. Western blotting was performed as described previously^36^. For each experiment, equal quantities of total protein were resolved by 10% sodium dodecyl sulfate–polyacrylamide gel electrophoresis (SDS-PAGE). The proteins were transferred onto polyvinylidene difluoride (PVDF) membranes (Millipore, Billerica, MA, USA). The blots were subsequently blocked in 5% skimmed milk and incubated for 24 h at 4 °C with the following primary antibodies: mouse anti-6X His tag monoclonal antibody (1:1000; Abcam), rabbit anti-FASN polyclonal antibodies (1:500; Abcam, Cambridge), rabbit anti-caspase-3 (1:500; Cell Signaling Technology), rabbit anti-cleaved caspase-3 (1:500; Cell Signaling Technology), rabbit anti-PINK1 (1:500; Proteintech) and anti-β-actin monoclonal antibody (1:5000; Sigma-Aldrich). After washing several times with TBS containing Tween (TBST), the membranes were incubated with horseradish peroxidase-conjugated anti-rabbit or anti-mouse IgG secondary antibody (GenDEPOT). The membranes were analyzed by chemiluminescence (Thermo Fisher Scientific and Bio-Rad, Berkeley, CA, USA) using a ChemiDoc™ Touch Imaging System (Bio-Rad).

### TUNEL staining

TUNEL-positive cells in lung tissue were detected using fluorescence microscopy (Nikon, Tokyo, Japan). The average number of dead cells in 20 fields per lung (n = 8/group) was assessed via the manual counting of TUNEL-positive cells in each high-power field (× 200).

### Immunofluorescence staining

For each paraffin-embedded specimen, 5 µm thick sections were baked, deparaffinized, and hydrated as described previously^38^. Specimen washing and antigen retrieval were done with sodium citrate solution. Sections were incubated overnight at 4 °C with primary antibodies : rabbit polyclonal FASN antibody (1:200; abcam), mouse monoclonal SPC antibody (1:100; Santa Cruz Biotechnology) and rabbit polyclonal PINK1 antibody (1:100, Proteintech). Antibodies were diluted in 1 × phosphate-buffered saline (PBS) with 1% bovine serum albumin (BSA) and 0.3% Triton X100. Secondary antibodies, Alexa Fluor 568 goat anti-rabbit immunoglobulin G (IgG) (H + L) (abcam) and Alexa Fluor 488 goat anti-mouse IgG (abcam) were used at 1:200 dilution. These were incubated for 2 h at room temperature. Nuclei were counterstained with DAPI stain solution. As a negative control, and to ensure specificity of staining, the primary antibodies were omitted from the staining procedure on some samples. Tissue were imaged using an Confocal Microscope (Leica, Germany). Images were processed using ImageJ 1.44 (WayneRasband, NIH, USA).

### Quantification of immunofluorescence positive cells

Following the double-immunofluorescence staining of FASN or PINK1 and SPC, each section was scanned using a Confocal Microscope (Leica). To quantify the double positive staining in the lungs, specimens were view at × 200 magnification, and ten fields per slide were randomly selected. These fields were digitally photographed in a blinded manner. The resulting digital images had thresholds applied manually. Next, the digital images were opened in Image J and the indices of fluorescence positivity were evaluated. The number of FASN positive only, SPC positive only, and both FASN and SPC positive cells were counted. The numbers of PINK1 and SPC double positive cells were also counted. Counting was performed by two independent observers for both IPF (n = 6), control human (n = 6), BLM (n = 6) and control mice (n = 6) samples. The numbers of each positive cells were is shown and expressed as mean ± SEM.

### Quantification of FASN intensities in AECs

To quantify FASN and SPC positive staining in the lungs, specimens were viewed at × 200 magnification, and eight fields per slide were selected in areas with a similar number of AEC cells. Fluorescence was demarcated using intensity-based threshold settings, and the number of particles was counted for each AEC cell in lung tissue using the particle analysis feature in Image J, following a modification of the previously described^39^. The total SPC fluorescence was quantified for each AEC cell in lung tissue, and FASN fluorescence that colocalized in SPC was calculated for each AEC cell. Counting was performed by two independent observers for both IPF(n = 6),control human (n = 6), BLM (n = 6) and control mice (n = 6) samples. The percentage fluorescence intensity relative to the control is shown and expressed as mean ± SEM.

### Apoptosis assay

FASN-overexpressing and knockdown cells were exposed to BLM (10 μg/mL), and mouse primary AECs were exposed to BLM (10 μg/mL) oleic acid (100 μM), palmitic acid (100 μM), and strearic acid (100 μM) overnight incubation in DMEM supplemented with 0.5% FBS. An Annexin V-fluorescein isothiocyanate (FITC)/propidium iodide detection kit (BD Biosciences Pharmingen) was used to determine the proportions of apoptotic and necrotic cells. Aliquots of approximately 1 × 10^6^ cells/mL were washed in PBS, surface stained, resuspended in binding buffer, and incubated with FITC-conjugated annexin V and propidium iodide (PI) for 15 min in the dark at room temperature, washed, and resuspended in binding buffer.

### Proliferation assay

Cell viability was measured using the WST-1 assay (Roche Diagnostics) in accordance with the manufacturer’s protocol. Primary mouse AECs were seeded in 96-well plates and incubated for 48 h. Then the cells were treated with BLM (3 mg/kg), BLM and 100 μM oleic acid, or oleic acid alone. WST-1 was added directly to the wells and incubated for 60 min at 37 °C. Then the plates were read by a scanning multi-well spectrophotometer by measuring the absorbance of the dye at a wavelength of 450 nm, with a reference wavelength of 630 nm.

### Generation of FASN transgenic mice and BLM-induced lung injury/fibrosis

Inducible human FASN transgenic mice were produced by the coinjection of SP-C-rtTA-hGH (courtesy of Dr. Jeffery Whitsett, Cincinnati Children’s Hospital Medical Center, Cincinnati, OH, USA) and pTRE-Tight-hFASN-His tag into C57BL/6 blastocysts (Orient Bio Inc., Charles River Technology, Sungnam, Korea) as described previously^9^. The FASN transgenic mice were given drinking water containing doxycycline (50 mg/mL; Sigma-Aldrich) to induce transgene expression. On day 0, the transgenic mice received BLM (3 mg/kg) in endotoxin-free water in a total volume of 100 µL by intratracheal delivery. Male FASN transgenic mice (8 weeks old) were randomly assigned to two groups (n = 8 per group). One group received drinking water containing doxycycline (50 mg/mL), beginning on day 0 until sacrifice on day 21, while the other group of BLM-treated transgenic mice received endotoxin-free water containing no doxycycline until sacrifice. As a control, FASN transgenic mice were treated with PBS (100 µL, intratracheally) on day 0 and housed with or without doxycycline-containing water (50 mg/mL) until sacrifice. At the end of the experimental period, bronchoalveolar lavage (BAL) was performed as described previously^40^. These mice were anesthetized using Zoletil (40 mg/kg) and Rompun (5 mg/kg) to induce general anesthesia in the animals, and the mice were euthanized by cutting the inferior vena cava.​ To investigate the preventive effect of oleic acid on BLM induced lung injury/fibrosis, eight-week-old male C57BL/6 mice were obtained from Orient Bio (Seongnam, South Korea). On day 0, the mice were administered 3 mg/kg BLM dissolved in a total volume of 80 µL endotoxin-free water by intratracheal instillation, oleic acid was administered immediately after bleomycin treatment by oral gavage at a dose of 3 mg/kg every 2 days for 3 weeks. To determine whether doxycycline had an anti-fibrotic effect, eight-week-old male C57BL/6 mice were administered BLM intratracheally and given drinking water with or without doxycycline (50 mg/mL) from day 0 until they were sacrificed on day 21. The experiments using mice were approved by the Committee on Animal Research at Soonchunhyang University Hospital (SCHBC_Animal_201609) performed following the relevant guidelines. All methods are reported in accordance with ARRIVE guidelines for the reporting of animal experiments.

### Masson’s trichrome staining of lung tissue

Lung tissues were fixed in Bouin’s solution, washed in tap water for 5 min at room temperature, and then stained for 10 min with Weigert’s iron hematoxylin. After washing in tap water, the slides were stained with a mixture of 1% acid fuchsin and 1% Biebrich scarlet in distilled water for 2 min, and then treated with 2.5% phosphomolybdic–phosphotungstic acid for 10 min. The sections were stained with aniline blue for 1 min, treated with 1% acetic acid for 1 min, and then dehydrated using a graded series of ethanol washes followed by five washes in absolute ethanol. Finally, the sections were immersed in xylene and then mounted in balsam.

### Measurement of hydroxyproline in the lungs of mice

To quantify collagen in the lungs, we performed hydroxyproline assays (Sigma-Aldrich) on the right lung of each mouse, in accordance with the manufacturer’s instructions. Briefly, the lungs were weighed, homogenized in sterile water, and hydrolyzed in 12 N HCl at 120 °C for 3 h. The hydrolyzed samples were incubated with 4-(dimethylamino) benzaldehyde for 90 min at 60 °C, and the absorbance of oxidized hydroxyproline was determined at 560 nm. The quantities of collagen present are expressed in micrograms per milligram of lung tissue.

### Mitochondrial membrane potential assay

BLM-induced changes in mitochondrial membrane potential (ΔΨm) were assessed using JC-1 fluorescence reagent (MP34152; Thermo Fisher Scientific) according to the manufacturer’s protocol. FASN-overexpressing/knockdown A549 cells were seeded in 6-well plates. Cells were treated with 10 μg/mL of BLM for 24 h. Mitochondrial membrane potential was measured by flow cytometry using the lipophilic cation 5,5,6,6-tetrachloro-1,1,3,3-tetraethylbenzimidazol-carbocyanine (MP34152; Thermo Fisher Scientific). Following BLM treatment, the cells were harvested and stained with 2 μM JC-1 dye in 500 μL HBSS/Ca/Mg buffer for 30 min at 37 °C. Emitted fluorescence in stained cells was detected at 580 nm (FL2 channel) using a flow cytometer (Becton Dickinson Biosciences, San Jose, CA, USA).

### Detection of mitochondrial superoxide

Mitochondrial superoxide was detected using fluorescent MitoSox-Red reagent (Invitrogen). FASN-overexpressing and knockdown cells were incubated in Hank’s balanced salt solution (HBSS) with 5 mM MitoSox-Red for 10 min at 37 °C in a 5% CO_2_ incubator. After removing MitoSox-Red and washing cells with HBSS, stained cells were excited at 510 nm under live confocal imaging and emitted fluorescence was detected at 580 nm (FL2 channel) using a flow cytometer (Becton Dickinson Biosciences, San Jose, CA, USA).

### LC–MS analysis of various fatty acids in FASN-overexpressing cells, knockdown cells and AECs from transgenic mice

Levels of palmitic acid (C16:0), oleic acid (C18:1)(9Z), stearic acid (C18:0), arachidic acid (C20:0), behenic acid (C22:0), lignoceric acid (C24:0) and linoleic acid (C18:2) were measured by liquid chromatography-mass spectrometry (LC–MS) in FASN-overexpressing cells, knockdown cells and AECs obtained from the transgenic mice. Cell pellets were homogenized, and samples were injected in a volume of 5 μL into an Agilent 1100 Series liquid chromatography system equipped with a degasser, autosampler, and binary pump (Agilent, Santa Clara, CA). Chromatographic separation was performed on a ZORBAX 300SB-C18 enrichment column (0.3 × 50 mm, 5 μm) at 35 °C with the temperature of the autosampler set to 4 °C. For the solvent system, mobile phases A and B were 0.1% formic acid in deionized water and acetonitrile, respectively. The mobile phase was delivered at a flow rate of 0.35 mL/min, and the entire eluent was carried into the mass spectrometer. The linear gradient was as follows: 0% B at 0 min, 25% B at 14 min, 100% B at 23 min, 100% B at 28.50 min, 0% B at 29 min, and 0% B at 35 min.

### Statistical analysis

Statistical analyses were performed using SPSS version 22.0 (IBM Corp., Armonk, NY, USA). The distribution of data was assessed using the Shapiro–Wilk test. The Student’s t-test was used to analyze continuous data. Normally distributed data were expressed as the means ± standard error of the mean. All P-values are two-sided and P < 0.05 was taken to indicate.

## Supplementary Information

Supplementary Figures.

## Data Availability

The datasets generated during and/or analyzed during the current study are available from the corresponding author on reasonable request.

## Abbreviations

AEC: Alveolar epithelial cell
BALF: Bronchoalveolar lavage fluid
BLM: Bleomycin hydrochloride
CoA: Coenzyme A
Doxy: Doxycycline
FASN: Fatty acid synthase
FBS: Fetal bovine serum
FITC: Fluorescein isothiocyanate
IPF: Idiopathic pulmonary fibrosis
OA: Oleic acid
PBS: Phosphate-buffered saline
PVDF: Polyvinylidene difluoride
PI: Propidium iodide
ROS: Reactive oxygen species
SPC: Surfactant protein C
siRNA: Small interfering RNA
