# Supplementary material for: Overexpression of fatty acid synthase attenuates bleomycin induced lung fibrosis by restoring mitochondrial dysfunction in mice
Source: Sci Rep. 2023 Jun 3;13:9044. doi: 10.1038/s41598-023-36009-3 (PMC10239437; doi:10.1038/s41598-023-36009-3)

Overexpression of Fatty Acid Synthase Attenuates Bleomycin Induced Lung Fibrosis by restoring mitochondrial dysfunction in mice

Running title: Overexpression of fatty acid synthase reduces lung fibrosis

Hyesun Shin[1*], Shinhee Park[1*], Ji su Hong[1], Ae-Rin Baek[1], Junehyuk Lee[1], Do-Jin Kim[1], An-Soo Jang[1], Su Sie Chin[2], Sung Hwan Jeong[3], Sung-Woo Park, [1†]

[1.] Division of Allergy and Respiratory Medicine, Department of Internal Medicine, Soonchunhyang University Bucheon Hospital, Bucheon, Korea.

[2] Department of Pathology, Soonchunhyang University Bucheon Hospital, 14584, Gyeonggi-Do, south Korea.

[3] Department of Internal Medicine, Gachon University of Medicine and Science, Gil Medical Center, Incheon, Korea.

[*] : Equally contributed as the first author

[†] **Corresponding author:** Sung-Woo Park, M.D., Ph.D.

Division of Allergy and Respiratory Medicine, Department of Internal Medicine, Soonchunhyang University Bucheon Hospital, 170 Jomaru-ro, Wonmi-gu, Bucheon 14584, Korea.

Tel: +82-32-621-5145

Fax: +82-32-621-6950

E-mail: swpark@schmc.ac.kr

Supplementary Figure 1.

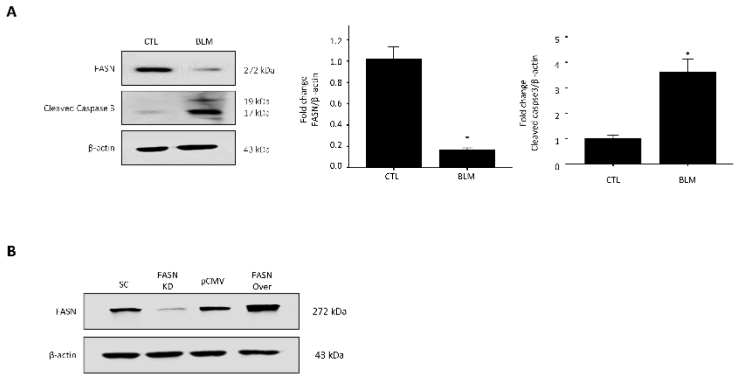

(A) Immunoblotting analysis of FASN, cleaved forms of caspase-3 in control and BLM(10 μg/ml)-treated AEC cells obtained from WT mice. The intensity of FASN and cleaved caspase-3 were quantified by densitometry, and the data were normalized to β-actin. The data are expressed as the mean ± SEM. Error bars represent mean ± SEM (n = 4, each group) *P < 0.05, vs. CTL. (B) Immunoblotting analysis of FASN expression in stable A549 cells (FASN_Over), FASN knockdown (FASN_KD), and their respective control vector-transfected (pCMV) and scramble control shRNA-transfected (SC) cells. Cropped images are displayed, uncropped blots are displayed in Supplementary Figure. 5.

Supplementary Figure 2.

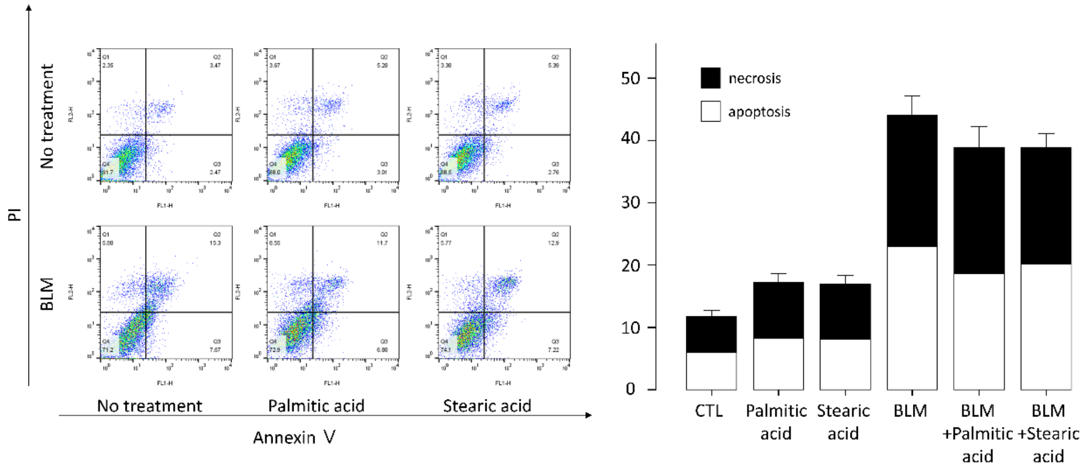

Treatment with Palmitic acid (100 μM) and stearic acid (100 μM) did not have a beneficial effect on AECs from BLM (10 μg/ml)-induced apoptotic cell death. Apoptosis was defined by Annexin V+/both PI+and PI−staining. Necrosis was defined by PI+/both Annexin V+and Annexin V−staining. The data are expressed as the mean ± standard error of the mean (n = 4). *<0.05 , BLM vs BLM + OA.

Supplementary Figure 3.

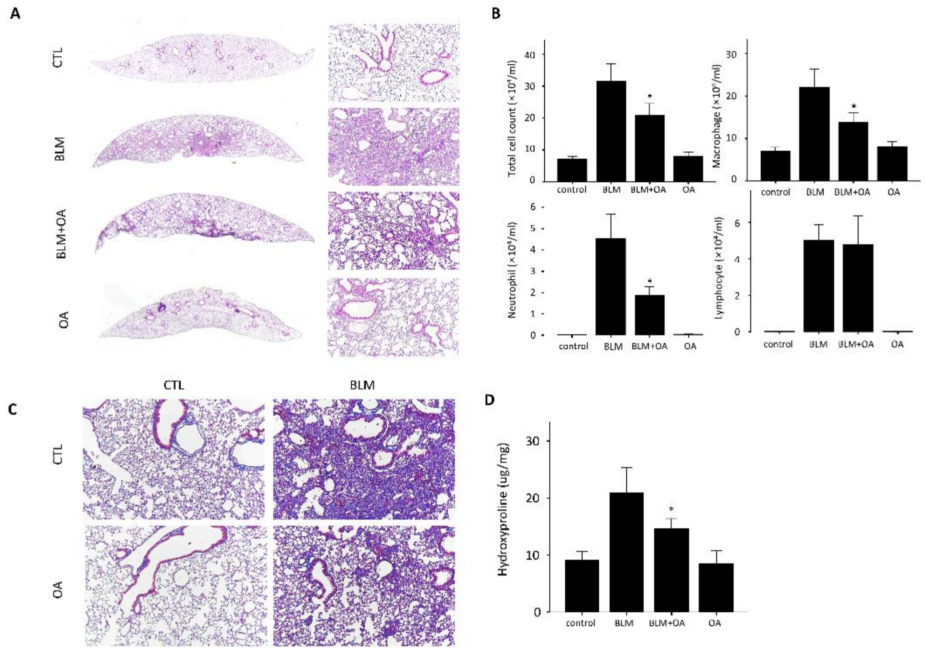

Treatment with oleic acid diminished BLM induced lung inflammation and fibrosis in mice. Oleic acid (3mg/ kg) was administered immediately to mice every 2 days after BLM treatment (3U/kg). (A) H&E staining of control, BLM, BLM +OA and OA -treated mouse lungs (n = 6 per group). Original magnification ×10, ; original magnification ×100 (B) Cell counts in BALF, collected on day 21. The total number of cells was counted using a hemocytometer. Differential cell counts in BALF were analyzed from 500 cells stained with Diff-Quick (n = 6 per group). (C) Masson's trichrome staining in the lungs of four groups of mice. Original magnification ×200. (D) Collagen measurement by the hydroxyproline assay in the lungs of four groups of mice (n = 6 per group).

Supplementary Figure 4.

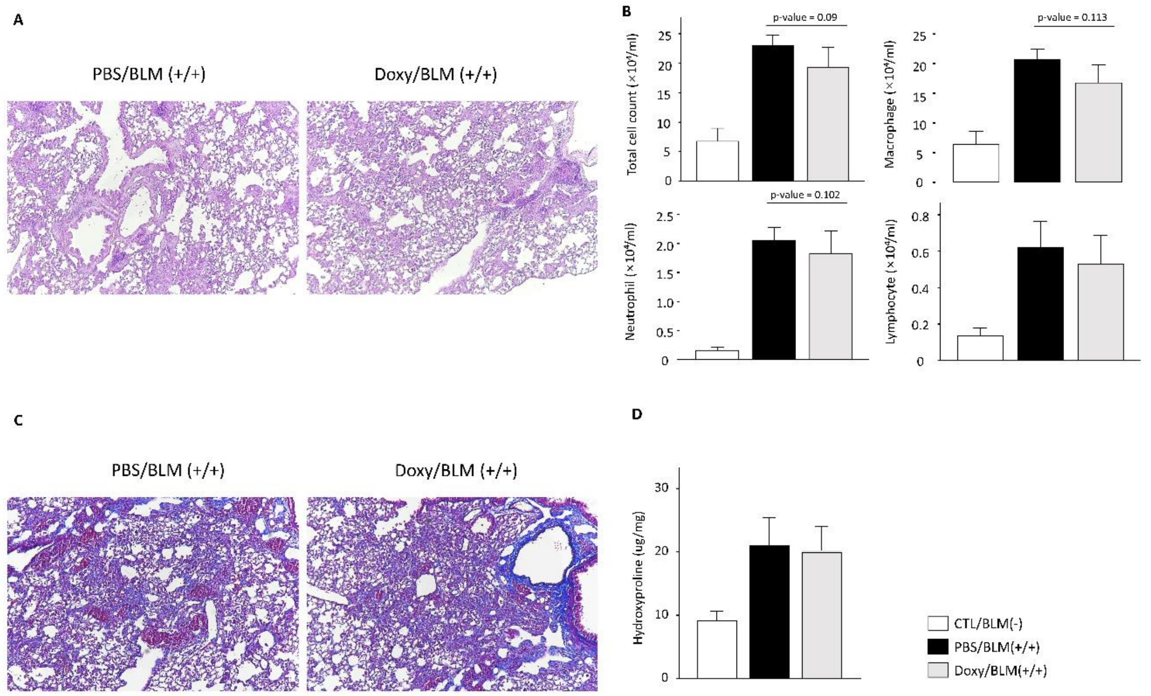

Doxycycline did not significantly diminish BLM-induced increased inflammation and fibrosis in the mouse lung. C57BL/6 mice (8 weeks, Male) were treated to BLM intratracheally with or without doxycycline-containing water for 3 weeks. (A) H&E staining of BLM-exposed mouse lungs with or without doxycycline treatment. Original magnification ×200. (B) Numbers of inflammatory cells in BAL fluid between doxycycline-treated and non-treated mice treated to BLM (n = 6 per group). (C) Masson's trichrome staining in the lungs of the mice. Original magnification ×200 (D) Collagen measurement by the hydroxyproline assay in the lungs of the mice (n= 6 per group).

Supplementary Figure 5. Uncropped blots from figure of the manuscript

Figure. 1B

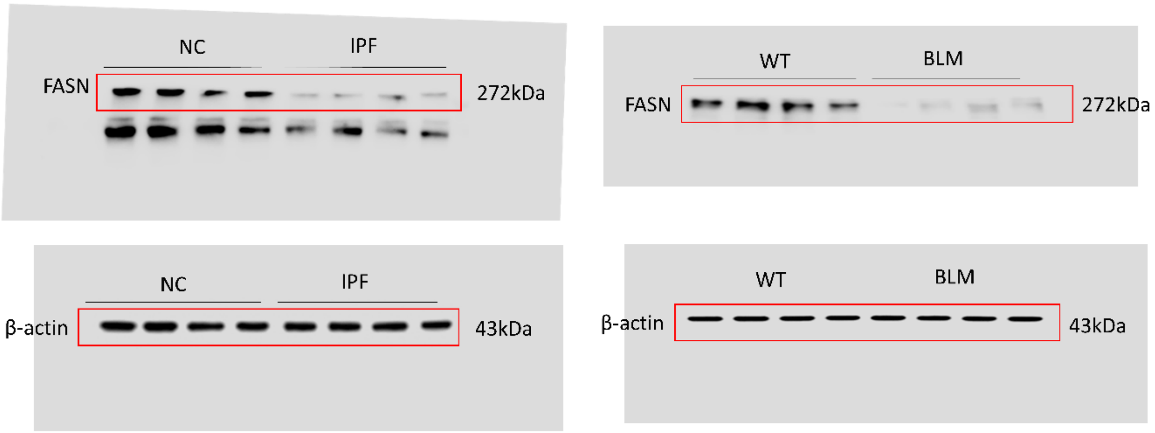

Figure. 2B

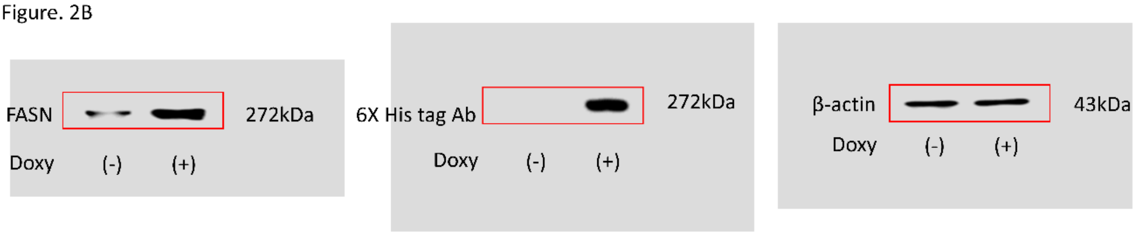

Figure. 3B

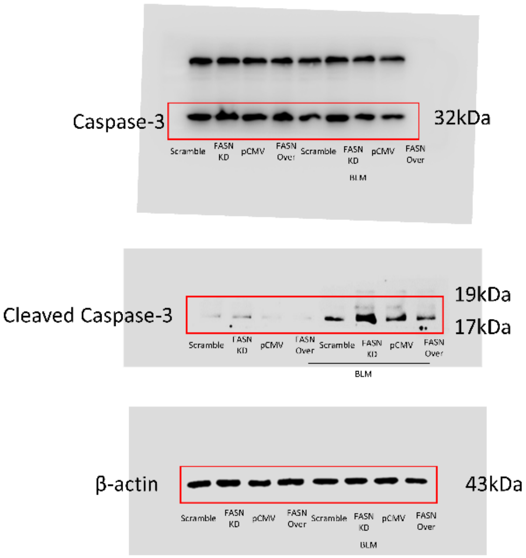

Figure. 4B

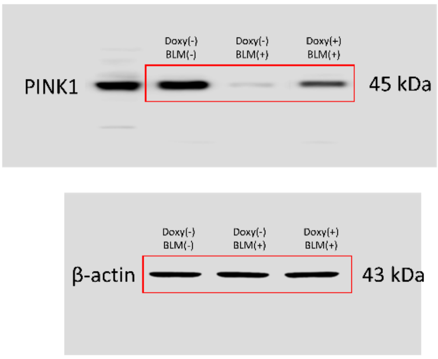

Figure. 7A

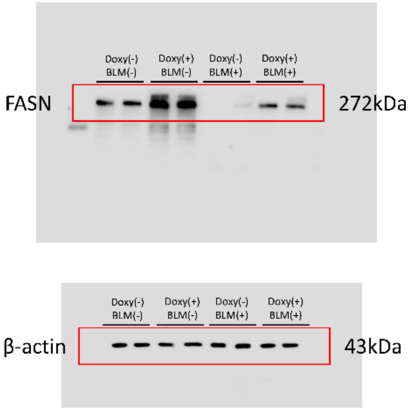

Supplementary Figure. 1A

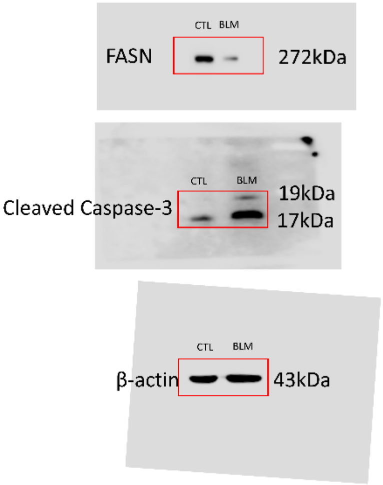

Supplementary Figure. 1B

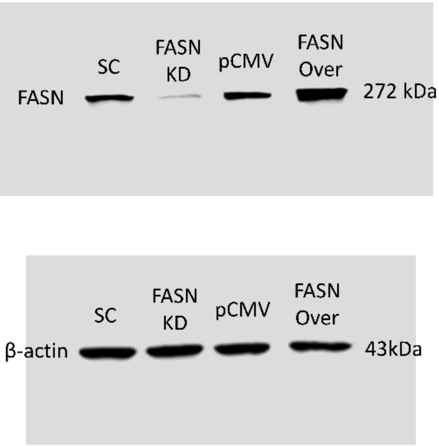

Supplement: Supplementary file 1 — Supplementary Figures. [file 41598_2023_36009_MOESM1_ESM.pdf]
